# Abstracts and Gleanings

**Published:** 1882-04-20

**Authors:** 


### ABSTRACTS AND GLEANINGS.

**Sexual Abnormalities.**—Dr. Ellis, in Baltimore Medical Association (Maryland Medical Journal) reported a case of congenital absence of uterus and vagina.

Dr. Ashby referred to a case in which there was no uterus; the ovaries were present. The lady had been married for several years and was well formed and handsome, with perfectly developed breasts. She was not aware of her deformity. The vagina was represented by a cul-de-sac about 1½ inch long.

Dr. Browne referred to two cases in which post-mortems had been obtained. The first, reported by the late Prof. Thomas R. Brown, had a mere cord in place of a vagina. The rudimentary representation of a uterus was discovered with the aid of the microscope. She had had the menstrual molimen regularly and vicarious menstruation. She was married—intercourse taking place through the urethra.

The second case occurred in the practice of Dr. Peaslee, who exhibited the specimens before a medical society. Battey's operation was performed in this case. A few muscular fibres were found representing one horn of the uterus.

Dr. Monmonier had had a case also of congenital atresia, not discovered until after marriage. The breasts were perfectly flat. Coition could not be affected, and the result was separation and divorce.

Dr. Stewart referred to two cases of atresia—one in a colored woman at the almshouse, in whom, at her own most urgent solicitation, an attempt was made to open a passage to the uterus. It was a most bloody operation, but was not successful. In another similar case a lady went to Edinburgh, was there operated on, and died in consequence.

Dr. Price related the following: A beautiful young lady, in a family whom he attended, became the object of the affections of a gentleman; his attentions were discountenanced by the parents, who were aware of her sexual malformation. The suitor being importunate Dr. P. was requested to make an examination with a view to informing the gentleman of her actual condition. He found a rudimentary penis, about one inch in length and the size of a goose-quill, through which she urinated. Beneath this little teat in the situation of the vulva there was no depression, nor even any sign of mucous membrane, the parts being smooth and covered with skin. The breasts were well developed; the voice that of a female—she sang beautifully. There were no testicles. There was evidence of the possession of erotic feeling. The mons veneris was well formed. Dr. P. informed the suitor of the result of the examination; he was nearly heart-broken by the news, and gave up business and moved away.

Dr. Johnston reported the following: He had been asked by the late Prof. E. Lloyd Howard, to see a case of apparently a young man, aged about nineteen. He had an effeminate look and weak voice, and dressed as a boy. This individual had come to learn his sex. A thorough examination was accordingly made. The mons veneris was distinct and covered with hair. There were apparent corpora cavernosa, and underneath these an apperture which communicated with the bladder. Under the aperture was a short cul-de-sac. No uterus was detectible. In each groin there was a gland-like body, believed to be a testicle. The conclusion arrived at was the individual was a male, and the explanation of the appearances found was traced in abnormalities of the fœtal development. Early in fœtal life the sex cannot be determined—the clitoris and penis being alike. If the two sacs holding the testicles remain separate, these organs may or may not descend. The spongy portion of the urethra and glans penis were absent in the case cited. The cul-de-sac might have been an excessively developed utriculus. This individual had passed for a girl up to the age of 18—associating constantly with them, and even *sleeping* with them. He even had long hair. At the age specified a thin beard began to show itself, when he cut off his hair and assumed the male attire. He had gone previously by the name of *Ann;* hence, to avoid unpleasant surprises, and at the doctor's suggestion he was baptized by the name of *An-drew.—Maryland Medical Journal.*

**Quinine.**—1. In the present state of our knowledge there are two modes in which antipyretic remedies may be conceived to operate: first, by increasing the discharge of the pyrexial heat; secondly, by checking its production.

2. The quantity of heat discharged may be augmented by direct withdrawal (tepid water), or by facilitating the circulation through the skin (digitalis, cutaneous irritants).

3. The production of heat may be lessened by repeated cooling of the surface, and especially by the internal use of antizymotics.

4. Febrile diseases commonly owe their origin to the introduction and rapid development of substances akin to ferments. Several of these have been shown to resemble yeast in being low vegetable organisms or derived from such organisms. They enter the glands, where they undergo multiplication, increase the metabolic process, generate products of decomposition which exert a paralyzing action on the nervous system, and raise the standard of temperature throughout the body.

5. Owing to impaired action of the heart in certain stage of the disorder, or to contraction of the cutaneous vessels, the skin becomes anemic and gives off less heat than usual. The internal temperature rises accordingly.

6. Quinine, our chief antipyretic, acts by directly combatting the efficient cause of the disorder, and by checking the abnormal metabolism going on in the body. The nervous system takes no part or only a secondary part in this operation. In intermittent fevers quinine prevents the paroxysms by attacking their infective cause. The paroxysms are not the essence—the substantive element—of the disease; they are only a symptom of it. The substantive element is the poison deposited in the colorless corpuscles of many organs, especially the spleen. There are fevers without paroxysms and paroxysms without fever. It is just those intermittent fevers which run their course without paroxysms that are the most malignant. The malarial poison rapidly causes disintegration of the tissues and the blood, and so paralyzes the nerve-centers.

7. The reduction of acute splenic tumors by quinine depends upon the adverse influence exerted by the alkaloid on the infective poison to which the morbid over-action of the spleen and its consequent enlargement are. "*Cessante causa cessat effectus.*" Even a healthy spleen may be reduced in size by large doses of quinine: the alkoloid vigorously checking the oxidation of its principal elements, the colorless corpuscles. Quinine has no direct influence on the vaso-motor nerves.

8. Quinine attacks the malarial poison with especial energy; on this fact depends the so-called specific action of quinine in intermittent fevers. The same relation, but in a minor degree, subsists between quinine and the infective poison of enteric fever, between mercury and iodine and the poison of syphilis, between salicylic acid and the "irritant" in acute articular rheumatism.

9. An antipyretic which in one disease instantaneously arrests the fever may be wholly powerless in another. The difference depends on the fact that the various antizymotics act very unequally upon the individual *schizomycetes* and ferments; one will paralyze rapidly, by another they will hardly be ejected.

10. The past history of the therapeutics and recent achievments in the domain of etiology and pharmacology entitle us to assume that by persistent scientific inquiry and practical observation we may succeed in discovering a specific antidote for every species of infective or septicemic malady.—*Prof. Binz, at Medical Congress.*

**Coated Pills.**—Of all the different methods of making pills tasteless by means of coating, the sugar and gelatine coating has been found to be the best. It has been said that pills must be dried out to allow a coating of sugar, and that is partly so; but drying out is necessary to keep the pills from spoiling and moulding. Suppose pills are coated while soft, they must be dried out *after* coating, else they will spoil and cannot be kept in stock.

If the coating of gelatine has to be done by means of needles, pills cannot be coated if they are hard, and therefore *must* be coated while soft, but must be dried out after coating; while pills to be coated with sugar can be dried out *before* coating, and thus do not need any drying out afterward.

If pills would be kept in stock without having been dried out, they would become mouldy and would spoil. We can have pills which remain soft and do not become mouldy, but such pills have been made by adding grease to the pill-mass. If the proper excipient has been used, hard pills may dissolve quicker than soft ones.

To prepare a pill-mass properly for making pills in large quantities, without resorting to means which should not be used, a long experience is required. Of such pills to be found in the trade, which have been properly prepared according to theory and practical experience, we should mention the pills prepared by W. R. Warner & Co., of Philadelphia.

These pills are soluble, can be kept in stock without spoiling, and are always of the same nice appearance and uniformity.—*Aptheker Zeitung.*

**Diagnosis Wanted.**—On Thursday, September 8th, I was called on by B. W. (aged 73). I found him suffering, as I thought, with simple diarrhœa. I left appropriate remedies, called next day, found him apparently well. Was sent for again on Saturday. The diarrhœa had returned; the stools were liquid and greenish in color, and there had been some vomiting, but very little pain; pulse 68, and temperature slightly elevated. Prescribed a simple diarrhœa mixture and ordered spice plaster to the stomach.

Was called at 3 a. m. Sunday. Vomiting all the time, stomach would hold nothing; diarrhœa excessive; pulse 106; temperature not much elevated; no pain. Tried to allay the vomiting, but did not succeed. Used opium and starch enemeta, and succeeded in partly checking the diarrhœa, but could do nothing with the vomiting. At 9 a. m. he appeared slightly better, vomiting only when disturbed, and bowels comparatively quiet. Had him to suck ice, and kept on with the injections of opium and starch, and spice plaster externally.

Made arrangements to consult with Dr. H. at 6 p. m., but was sent for again at 5; found him in a state of collapse; had been that way for three hours. Gave whisky in small doses frequently till 6 p. m., when Dr. H. arrived. He advised the administration of whisky in heroic doses. We gave him large doses 'till 9 o'clock, but could make no impression. At 9:10 he vomited freely, pure bile, and again at 11, and kept on with whisky till 12. Even this appeared to have no stimulating effect, and patient died at 2:15 a. m.—*Monthly Review.*

[There is reason to believe that there are rare instances in which the liver secretes an acrid, poisonous bile which produces emesis and violent cramps and colics, and in some instances death. In such cases the diarrhœa should not be checked, but a purgative treatment adopted to assist nature in throwing off the acrid matter.—W., ED. RECORD.]

**The Disadvantages of Cod-Liver Oil for Young Children.**—According to the *Revue Medicale*, the Council of Public Health has recently submitted for the sanction of the Academy of Medicine of Paris a report on the disadvantages of cod-liver oil administered to infants and young children. The commission on the hygiene of infancy has not yet reported its opinion on this subject; but the accusations brought against this medicine by the Council of Hygiene are worth notice. All physicians are aware what disastrous influence is exercised on the health of young infants by defective administration, and especially animal nourishment; fatty matters are as little suited to the alimentation of newly-born infants as albuminoids, excepting always casein, which exists normally in milk, and is found to be perfectly assimilable. In fact, in the first period of life, the juices necessary for emulsifying fatty matters are almost entirely wanting. The liver, in spite of its enormous development in this stage of existence, secretes only a small quantity of bile; and the researches of Langendorf and Zweifel have proved that, in young children, pancreatic juices and emulsive power is almost nil, or, at least, very slightly marked. These physiological considerations sufficiently indicate that—far from being profitable to the infant—fatty matters, and especially cod-liver oil, can only injure its health, and gravely compromise the integrity of its digestive functions.—*British Medical Journal.—Medical Weekly.*

**Sabal Serrulata—Saw Palmetto.**—This plant, a native of Georgia, Florida and South Carolina, has within the past few years been brought prominently before the physicians of this city and State by Dr. James B. Read, and owing to so little having been written about it, or on its medicinal properties, it is hardly known beyond the borders of Georgia as a most valuable remedial agent.

The preparations are various, the "Saccharated Oil," "inspissated juice," and the "Elixir"—its taste is sweetish and butyric. The "Saccharated Oil (a sugar) is a pale canary color and very light in weight; the inspissated juice, about the consistency of malt, and the Elixir resembles clear or amber colored syrup. All of these preparations are of a very pleasant flavor, and I may say palatable, so much so that little children and nervous women take any of them with ease.

Among the medical properties and uses of the Saw-Palmeetto I find prominently its soothing effects on the mucous membranes, producing a very pleasant sensation on the throat and fauces, relieving soreness, hoarseness, coughs, catarrh, coryza and colds in the head; encourages sleep by its soothing sensation—if taken for any length of time it increases weight and produces fat.—*Dr. Myers, in Monthly Review.*

**A New Method of Treating Endometritis.**—Dr. S. S. Boyd, of Dublin, Ind., in a communication to the American Practitioner, for October, 1880, says—

Within three years I successfully treated, by a method original with me, a very obstinate endometritis, occurring in a woman twenty-five years of age, who had been married five years. During all of her married life, until recently, she suffered from a constant flow of muco-purulent discharge from the uterus, with all the attendant symptoms of endometritis. Much of the time she was scarcely able to walk about the house. During the two and a half years which I treated this patient, I exhausted all of what I considered safe remedies, both topical and general, with but little benefit. Finally, I adopted the following plan of applying nitrate of silver to the endometrism:

Taking a small female silver catheter, I had it cut in two, so as to leave three inches of the closed end in one piece. In three-fourths of an inch of this closed end I had as many small perforations made as could be, without materially weakening the walls of the instrument, and to the outside of the open end a ring was soldered, to which a small cord could be attached. Having on the day previous to that on which I used this instrument introduced into the uterus a slippery-elm tent, retaining it in place by a pledget of cotton wool, I let the tent remain over night. Putting about fifteen grains of coarsely pulverized nitrate of silver in the tube above described, and confining it there by pressing a little cotton on it, I then tied a small cord, six inches long, to the rim, when it was ready for use. Removing the plug and the tent after introducing the speculum, I inserted the silver tube into the uterus, until the distal end reached the fundus, securing it in place as I did the elm tent, leaving one end of the cord outside of the vagina. This was done as a precaution against any serious pain in my absence, in which case the patient could remove the tube. But it was not found necessary to remove the instrument for three or four hours, and then the nitrate of silver was dissolved.

Briefly, the foregoing treatment was that which finally relieved a long suffering patient, in less than six weeks, by four applications one week apart. Of course, I did not neglect to administer iron, sulph. quinia, ale and ext. malt, as I consider constitutional medication in such cases essential to the relief of the local disease.

As this mode of topical application to the internal uterus was tried in but a single instance, no certain deduction can be drawn as to its general adaptation to the cure of endometritis, and yet, from its complete and speedy success in this single case, I am led to hope it may prove a valuable addition to the local treatment of this form of uterine disease.—*Med. and Surg. Rep.*

**Nitrite of Amyl in Convulsions.**—Dr. Leonard F. Pitkin, of Ravenswood, Long Island, reports the following case in the Medical Record, October 2d, 1880—

Willie R., aged two years and six months, while at play on the morning of August 12th, was suddenly seized with convulsions. I was immediately summoned, and on my arrival I found the child in an unconscious state, one convulsion following another in most rapid succession. The convulsive movements were confined almost entirely to the right side. My first impression was that the convulsions were due to functional derangement of the stomach and bowels, and the usual remedies indicated in such cases were resorted to, but no benefit followed the same. As the child was, in the meantime, rapidly becoming exhausted, and the necessity of doing something to control the convulsions more evident, I resorted to the use of chloroform and ether, but without succeeding in controlling the convulsions. I then used nitrite of amyl by inhalation. Placing four drops on a handkerchief, I applied it to the child's nostrils, and after a few moments had passed the convulsions ceased entirely, and did not occur again till yesterday morning, August 13th, but were immediately controlled by four drops of amyl nitrite, and have not occurred since. A close examination of the patient reveals that the convulsions were evidently due to some cerebral lesion. The cranium is markedly symmetrical; pupils unequally dilated, the left being much larger than the right. The head is not abnormally large, but the posterior fontanelle is perceptible, union not having taken place between the bone, and remarkably large, being an inch and a quarter in diameter at its base. There is evidently some serous effusion in the membranes of the brain. Child is now taking—

℞ Potass. iodidi .................................... gr. x.

Brom. potass ...................................... gr. xl.

Aquæ, ...................................... } aa ℥ j.

Syr. rhei., ...................................... }

Sig. Teaspoonful t. i. d.

The child is well nourished; appears strong and healthy. On questioning the mother, I ascertained that she has lost two children, both dying in convulsions, which were like this one. I have used the amyl nitrite in a large number of cases, and, with few exceptions, it has been followed with good results. I usually administer five minims, dropping it into a small sponge, allowing the patient to inhale it. It causes the face to flush, and stimulates the lachrymal glands to a considerable extent. Great care should be used in the administration of the drug, as serious consequences might result from its injudicious use.—*Med. and Surg. Rep.*

**Chloral and Bromide of Ammonium in Febrile Delirium.** —Dr. C. H. Hughes (*St. Louis Medical and Surgical Journal*) says:

"An extensive experience with these therapeutic agents in the delirium of fever justifies its confident commendation to the practitioner of medicine; an experience begun many years ago at Fulton, with their use in the delirium of mania, and extended there and elsewhere to a delirium associated with all other forms of disease, from that of typhoid and the exanthemata to delirium tremens and aggravated hysteria. In fact, no drug, in hysteria, equals a full resistless dose of chloral, the patient usually awakening from her "tantrum," refreshed, rested and tranquil in her nerve-centres, which for hours before were all unstable and unstrung.

"The true therapeutic principle in the use of these valuable agents is tranquilization and the recuperation and resistance to decay which the restraint exerted by them brings about. The ammonium bromide for use during the day, and the chloral once only at night. Twenty to thirty grains of the former, *ter in die*, and as small a dose of the latter as wlll induce sleep at night, and largely diluted with water, milk or beef-tea, the beef-tea being preferable in all typhoid states.

"While large doses of chloral are indicated in maniacal excitement, in febrile delirium only small doses are required.

"To periodically arrest cerebral disintegration in febrile delirium, at the natural time for sleep, is a point gained each day in the direction of restoration, as shown in the often apparent improvement of the patient after each waking, and enables the *vis medicatrix naturæ* to better fight the battle of life with the destructive disease."—*Med. and Surg. Reporter.*

**Tetanus Cured.**—Dr. Layton, in the New Orleans Medical Journal, reports a case of tetanus cured with the following prescription:

℞ Sulphate of eserine.......................... gr. ½,

Pure glycerine.............................. f. ℨij,

Syrup of orange flower,...................... f. ℨxiv,

Water..................................... f. ℥ij.

M. S. Teaspoonful (1-64 grain or one milligramme of eserine) every hour. I should say here that I was advised by Mr. Lascar, the obliging Chemist of Messrs. Lyons & Co., to use the solution in glycerine, eserine being so easily decomposed otherwise. Even the short exposure to the air of the salt, during the time required for preparing a dose, is sufficient to cause an increase in weight of the eserine so exposed. Mr. Lascar has remarked that glycerine prevents the decomposition of the solution.

From January 10th, in the evening, the doses of eserine were given at intervals of an hour and a half; later, the time was increased to two hours; the remedy was continued until January 17, when the child had taken, in all, 3 grains of eserine; the prescription was then discontinued, the only remaining trace of the attack being some rigidity of the jaws, which had entirely disappeared by January 30th.

[Eserine or physostigmine is an alkaloid obtained from the calabar bean, and is an agent of great power and should be given with caution.—ED.]

**Bovine Lymph.**—Dr. Morris, in Maryland Medical Journal, remarks:

First. That the healthy heifer, innoculated with pure, spontaneous cow pox, supplies a vaccine lymph which, when introduced into the human economy, produces all the symptoms to be found in vaccination by human lymph, but in a more marked degree.

Second. That the protection afforded by vaccination with bovine lymph may be presumed to be fully equal to that obtained by human virus, but this fact cannot as yet be proved by statistics.

Third. That the dangers from human lymph are greatly exaggerated, and, if they exist at all, may have a counterpart in the animal economy.

Fourth. That cow pox, transmitted through heifers, is more active and more violent in its effects than human virus, and, if these evidences are a proof of the efficacy of the vaccination, it must afford perfect immunity from small-pox.

Fifth. That, by the use of bovine virus, we can at all times have an ample supply of fresh lymph, a consideration of much weight in the event of an epidemic.

Sixth. As human lymph produces its action on the system at an earlier period than bovine lymph, it would appear to be better to use it in those cases in which vaccination is employed as an abortive agent.

Seventh. That further investigations are necessary to establish the true character of bovine lymph, and it is the duty of the profession to collect and publish all the facts bearing o[illegible] the subject.

**Treatment of Ozæna.**—Dr. Cozzolino, of Naples, has recently written a monograph on this subject. He recommends a pomade as follows:—

| | | |
|---|---|---|
| R. | Hydrarg. chlor. mit | grs. xxx. |
| | Sodii benzoat | ℥ iiiss. |
| | Sodii salicylat | gr. xv. |
| | Thymoli | gr. j. |
| | Iodoformi | ʒ j. |
| | Ung. petrolei | ℥ ss. |
| | Acidi tannici | gr. j. |
| | Rosar. essentiæ | ʒ j. M. |

This to be applied locally, after detersive injections, by Weber's nasal douche.

He recommends prudence in the application of mercurials, and attaches particular importance to a general anti-scrofulous treatment.

For ozæna from atrophic rhinitis he recommends benzoic acid, certain mineral waters in douches or in powder, such as the water of Saint Christian, justly extolled before him by Dr. Tillot, and that of Casamicciola, etc.

He is no partisan of the tampons of cotton-wool of Gottstein, and prefers to them medicated bougies of gelatine of a form invented by himself—a conical form and 3, 4, or 5 centimeters in length, adapted to the calibre of the nasal fossæ. The object of these gelatinous bougies is that they may remain upon the diseased surface in order to obtain their full action. They are made up with the following ingredients.

1. *Astringent or Anti-catarrhal Gelatinous Bougies.*—Subnitrate, tannate, and salicylate of bismuth, pure tannic acid, sulphate of zinc, and sulpho-carbolate of zinc.

2. *Emollient or Solvent Gelatine.*—Chloride of sodium, chlorate of potash, chloride of ammonium, neutral alkaline carbonates, employed to dissolve the inspissated secretions in some cases of dry rhinitis.

3. *Modifying or Resolving and Specific Gelatines.*—Preparations of iodine and of mercury, as, for example, iodoform, calomel, or red precipitate. For specific cases, the sulphur in herpetic lesions, and corrosive sublimate in syphilitic affections.

4. *Anti-fetid or Disinfecting Gelatines.*—Vegetable charcoal, thymol, salicylic acid, and phenol.

The washing ought always to precede the application of the gelatines and the insufflation of medicated powders. The gelatines are applied alternately in each nasal fossa, especially in the evening. In the morning we ought to introduce the bulb of the nasal douche in the opposite nostril to that in which the gelatine had been applied the previous evening.—*Medical and Surgical Reporter.*

**The Danger of Administering Anæsthetics Without Witnesses.**—This subject has been written upon time and again, and physicians have been repeatedly warned not to give an anæsthetic to any one, but in an especial manner to avoid using them with females, unless in the presence of witnesses. Yet the advice is unheeded, and every now and then we hear of some unfortunate results from this negligenee. This time it comes from the West. An old and prominent physician was visted by a beautiful young lady, for the treatment of some sexual disorder. The lady belonged to the blue blood of the city, while the physician had been her grandparents' attendant, and had for years been the family physician. On one of her visits he informed her that an operation was necessary, and induced her to take some anæsthetic Subsequently, not improving, she consulted another physician, who told her that she was pregnant. In due time a child was born.

The lady made an effort at concealment, but in a short time brought an action for damages against her old doctor. He publicly denied the charge, but refused to go into court and do so under oath. After a few minutes' deliberation only, the jury brought in a verdict against the doctor, assessing the damages at fifty thousand dollars. The verdict was received with such open manifestations of approval from the judge, as well as the audience, the former publicly shaking hands and congratulating the young lady in court, as to leave no doubt of the sympathy of the public. The case is a sad one; whether guilty or not, this old and established physician has been ruined, socially and professionally, forever.

It should be made a rule, never to be broken, by every physician, when he commences the practice of medicine, to refuse absolutely to administer an anæsthetic to any woman, alone, no matter what the collateral circumstances may happen to be.

Women are peculiar and unreliable, and there is no accounting for the queer notions they may sometimes get into their heads; so that no physician is surely safe from trouble who has passed a period alone with a woman who has been made unconscious by an agent administered to her by him.—*Medical and Surgical Reporter.*

**Case of Poisoning by Atropia.**—The patient, a small, weak, anæmic child, aged 6, took by mistake, between 8 and 9 in the morning, a teaspoonful of a solution of atropia, 0.05 to 10.0, or about 35 milligrammes (a little more than half a grain). His face soon became flushed, his gait staggering, and his voice hoarse. An hour later antidotes were administered, including tannic acid, iodide of potassium, and injections of infusion of jaborandi. Soon the patient exhibited hallucinations, and became delirious. Seven and a half hours after the dose had been taken, Reinl (*Prag. Med. Wochenschr.*, No. 20,1880) saw the child, who was restless and unconscious and still delirious, the pulse being 140 and the respirations 30. A hypodermic injection was given of 5 centigrammes of morphia (about three-fourths of a grain), and in ten minutes the pulse fell to 100 and the respirations to 20. The restless movements subsided, and in half an hour the patient slept, the pulse falling quickly to 96 and the respirations to 18. The following morning, after a good night's rest, the child was quite cheerful, though there remained twitchings of the muscles of the face and extremities which continued three days. [This case affords no proof that morphia is an antidote to atropia. Children are curiously insusceptible to the action of belladonna. Holthouse has recorded the case of a boy, aged 3½ years, who recovered after taking half a grain of atropia; there have been cases of recovery after taking 0.5, 0.6, 1.0, and 1.5 grains respectively. The injection of an infusion of jaborandi could hardly be expected to do any good. It is true that a hypodermic injection of a one-hundredth part of a grain of atropia will instantly check the perspiration and salivation of jaborandi, but the converse is not the case. Atropia is a much more powerful alkaloid than pilocarpin; and in a case of poisoning by atropia recently recorded by Purjesz, of Buda-Pest, as much as 6½ grains of hydrochlorate of pilocarpin had to be administered hypodermically, before any antidotal properties were observed. It is interesting to note the fall in pulse and respiration after the injection of the morphia. It would appear that, although morphia is not an antidote to atropia, it will retard the restlessness and delirium caused by a poisonous dose of that alkaloid.—*London Med. Record.*]

**Physicians who do not Read.**—Whether they are too busy, or too illiterate, or that they are not yet beyond the influence of their earlier habits and associations, it seems to be a fact, if implicit reliance can be placed on a recent statement in the columns of our cotemporary, the *St. Louis Medical and Surgical Journal*, that scarcely one-half of the 26,000 physicians practicing in the western and southwestern States take a medical journal of any kind. Of these, one-half, it states, take the cheapest medical journal they can obtain, so cheap, indeed, that it will be sent to them whether it is paid for or not. To this sweeping assertion there are numerous and marked exceptions, and we recall with pride and pleasure the names of many excellent members of the profession in those sections who have adorned its practice or its literature by moral and mental gifts of a superior order. There are hundreds, however, who have never studied medicine, attended lectures or taken a medical degree from any school. They have graduated themselves from the butcher's block or the tailor's bench, because they had no qualifications even for those branches of trade; and a few days after deserting a locality which they could not embelish, they have reappeared at some other point, fully licensed as medical practitioners (by their own act), to take the lives of an unfortunate community into their keeping. Is it any wonder that such men do not read a medical journal, or open the pages of a professional treatise, for information which is far above their comprehension!—*Clinical Record.*

**Codeia in Dysmenorrhœa, Sleeplessness and Malarial Headache.**—Case I. I was consulted by the mother of a young lady of 18 years who, she stated, had suffered with painful menstruation for the past two years. The pain was so great that she neither got rest nor sleep during her periods. After putting her under a general medical treatment, I ordered opium to relieve the pain, but it disagreed with her sensitive stomach. Morphia was no better, though I used it hypodermically. It was then I decided on codeia, in one-fourth of a grain doses, and had the pleasure of seeing my patient perfectly relieved. Her pain disappeared and a calm sleep was induced. From this happy result I decided to try it on another case, where morphia had played a prominent role, and had failed.

CASE II. A lady 35 years, unmarried, was subject to dysmenorrhœa to such an extent, that she had to keep her bed during four days of her catamenia. I ordered codeia, in one-fourth of a grain doses, morning and evening, with prompt relief.

CASE III. Married lady, 40 years old, complains of distressing pain during her catamenia. About two years ago she aborted at fourth month, and had suffered to a greater or less extent ever since, at her regular returns. Physical examination showed an ulcerated os and an anteflexed womb. While treating the last two affections, I administered codeia to relieve her pains with the same unfailing and pleasant result.

Encouraged by these experiences, I prescribed it in a case of mania-a-potu, and in twenty minutes my patient was calmed, and upon the repetition of the dose he fell asleep.

Again in a case of great exhaustion, in a gentleman who had to take twenty grains of hydrate of chloral, with one drachm of bromide of potassium, in order to obtain an hour's sleep, I ordered one-fourth of a grain of codeia, to be repeated in twenty minutes, and for the first time in two months that gentleman enjoyed four hours of unbroken sleep. I have used it also in the distressing headache that accompanies malarial fever, and always with the most flattering results.—(*College and Clinical Record.*)—*Obstet. Gazette.*

**Eczema versus Vaccination.**—On page 144 of current volume we reproduce a statement by Dr. Murray, in the *British Medical Journal*, to the effect that vaccination exercises a very salutary influence on the course of eczema as occurring in infants. He refers to his experience in support of the statement, and confidently appeals to medical men of middle age for facts in their practice in further corroboration. So far from deferring vaccination, as is usually done, because of the existence of an eczematous eruption, he regards such existence as an additional reason why the child should be vaccinated.

And now comes Dr. George Thin, of London, (*Edinburg Med. Journal* for December, 1881) who gives a synopsis of the discussion on the subject of vaccinal skin-eruptions before the late International Medical Congress, and supplements the same with his own personal experience, the inference from all of which, viz., that vaccination may develop eczema, raises *inter alia*, a very interesting question in therapeutics. In the discussion referred to Prof. Hardy, of Paris, divided vaccinal eruptions into four kinds—

generalized vaccination, exanthematic eruptions and diathetic eruptions (eczema, etc.) The second of these occur before the development of vaccine, and the last after the development of vaccine, of the vaccine pustule. All practitioners of experience have observed the occurrence of eczema after vaccina'ion, and have probably at times been hard driven for such an explanation as would satisfy the parents as to the purity of the virus.

The debate before the Congress leaves no doubt that eczema follows vaccination with virus which is above all suspicion of impurity, and when this fact is placed beside the statement by Dr. Murray that vaccination is the very best remedy for idiopathic eczema, have we not an illustration of the law of *similia similibus?* —*Michigan Medical News.*

**On the Real Position of Rotheln, Bubeola or German Measles.**—W. Squire, M. D., London, in Medical Congress, gave a short historical survey of the literature of this disease, and showed that it was known before it received a distinctive name. He said that the disease, in his opinion, had but a superficial resemblance to scarlet fever, but had close relations to measles in several points. But it was self protective, was as distinct from measles as varicella from variola, and possessed all the marks of a specific disease. It was contagious, it ran a definite course, and it occurred but once in the same person.

Dr. Kassowitz, Vienna, said that in the epidemic of rotheln which had come under his observation he had never noticed the affection passing into measles. The resemblance to measles was nevertheless sometimes so marked, both as regards the eruption and the associated phenomena, that in any single case the distinction from the milder form of measles, which ran a rapid course, was rendered extremely difficult, and, in such circumstances, could generally only be made by having regard to other cases in the same house and family. If this affection had any special relationship, it was to measles, not scarlet fever.—*Med. and Surg. Reporter.*

**The Alum Plug in Uterine Hemorrhage.**—Dr. R. W. Griswold, Connecticut, says:

For the last twenty years my reliance has been on a junk of alum in the vagina. If this is not at hand I take the next best thing that is; but a junk of alum is a part of the contents of my medicine box. It is of the size of a large hen's egg, ovoid in shape, and generally left a little ragged, though without sharp points. Around the middle is cut a groove, about which is tied a bit of strong but not large twine, leaving the ends so that they can hang out of the vagina. This treatment is easy, speedy, and effectual against further hemorrhage. It has never failed me, and I leave a patient with a feeling that she is safe for the next twelve or fifteen hours, so far as danger from further bleeding is concerned. And I may add that I have never had any unfavorable effects follow its use in any one of the scores of cases in which it has been employed—no fevers, no septicemia, no deaths, no anything ontoward—and I have never had occasion to use it the second time in any one case.—*Western Lancet, San Francisco.*

**Antiseptic Inhalation.**—Dr. Jos. O. Hirschfelder, of California, (in Pacific Medical and Surgical Journal,) says:

We are to search for the remedy for consumption in the form of a volatile antiseptic. No one of greater power than those familiar to us has as yet been found, but a better method of their administration has been discovered and described by Curshmann, of Berlin. He employs a respirator, having a wire gauze chamber filled with cotton, upon which the substance to be inhaled is poured. Since his description of the apparatus it has been extensively employed in Germany and England, and, inasmuch as the reports have been highly favorable, I had the instrument made, with some slight modifications, by Folkers Bros., of this city. The apparatus consists of a mouth piece, in the center of which a wire gauze chamber is found. In this chamber absorbent cotton is placed, and upon the latter the desired remedy is poured. I have employed oleum terebinthinæ, oleum pini rectificatum, creasote, and carbolic acid. The results that I have obtained have been most encouraging. The sputa have diminished most perceptibly and the fever has been markedly reduced under its use.

**Explosion of Aqua Ammoniæ.**—The *Pharmaceutical Journal* records a recent case of an explosion of ordinary liquor ammoniæ followed by serious results. A Belfast woman, subject to headache, sent her daughter to the druggist to purchase a small quantity of "head salts," for which he gave her liquor ammoniæ, or "spirit of hartshorn." instead of the salt, carbonate of ammoniæ. The vial was put on a shelf and not used for a few days. Having a headache, the woman lifted the remedy to apply it, and had it in her hand for a few minutes only when the vial suddenly exploded, scattering the contents over her face. Her eye was destroyed, and her mouth and throat burned, the skin of both having been torn off. The vial had been put on the mantelpiece previous to the time it was used, and when about to apply the contents the woman was sitting near the fire.—*American Med. Weekly.*

**Prophylaxis of Small-Pox.**—Dr. Ellis, after stating that he was now attending a case of small-pox, asked for the best means of preventing its communication from the patient to others with whom he came in contact.

Dr. Taneyhill spoke of the method which he had employed in 1869. He always saw the patients after meals; he talked through a handkerchief whilst in the room, and he always changed all his woolen clothes in an adjoining room before entering that occupied by the patient.—*Maryland Med. Jour.*

**Parsley as an Antigalactic.**—Dr. Martin (*Bull. gen. de Therapute,*) states that if the breasts of a nursing woman be covered with parsley leaves freshly pulled, the application being renewed several times a day, as quickly as the leaves fade, the milk will soon cease to appear. This is an application which may be used when it is impossible to give purgatives or other remedies internally.—*Med. and Surg. Reporter.*

